# A Polypeptide of Tumor-Associated Antigen L6 with Intrinsic Adjuvant Activity Enhances Antitumor Immunity

**DOI:** 10.3390/vaccines8040620

**Published:** 2020-10-21

**Authors:** Yuh-Pyng Sher, Kit Man Chai, Wen-Ching Chen, Kuan-Yin Shen, I-Hua Chen, Ming-Hui Lee, Fang-Feng Chiu, Shih-Jen Liu

**Affiliations:** 1 Graduate Institute of Biomedical Sciences, China Medical University, Taichung 404, Taiwan; ypsher@mail.cmu.edu.tw (Y.-P.S.); j97013537@gmail.com (W.-C.C.); 2 Chinese Medicine Research Center, China Medical University, Taichung 404, Taiwan; 3 Research Center for Chinese Herbal Medicine, China Medical University, Taichung 404, Taiwan; 4 Center for Molecular Medicine, China Medical University Hospital, Taichung 404, Taiwan; 5 National Institute of Infectious Diseases and Vaccinology, National Health Research Institutes, Miaoli 350, Taiwan; ckm0509@nhri.edu.tw (K.M.C.); shenky057@nhri.edu.tw (K.-Y.S.); 007yvonne@nhri.edu.tw (I.-H.C.); estilee@nhri.edu.tw (M.-H.L.); cherrychiu@nhri.edu.tw (F.-F.C.); 6 Graduate Institute of Medicine, Kaohsiung Medical University, Kaohsiung 807, Taiwan

**Keywords:** TAL6, lipoprotein, TLR2

## Abstract

Peptide vaccines are safe, and aim to elicit and expand tumor-specific immunity so as to eradicate tumors. However, achieving strong and long-lasting anti-tumor immunity with peptide vaccines for the antigen-specific treatment of cancer is challenging, in part because their efficacy depends on strong adjuvants or immunomodulators. We approached this problem by conjugating an epitope-based cancer vaccine with a lipidated sequence (an immunomodulator) to elicit a strong immune response. Lipidated and non-lipidated polyepitope proteins were generated that contained the universal T helper cell epitope (pan-DR), B cell epitopes, and the extended loop sequence of extracellular domain 2 of tumor-associated antigen L6 (TAL6). We show that the lipidated polyepitope cancer vaccine can activate bone marrow-derived dendritic cells, and trigger effective antigen-specific antibody and T helper cell responses, more effectively than the non-lipidated vaccine. Moreover, potent T cell immune responses were elicited in mice inoculated with the lipidated polyepitope cancer vaccine, providing protective antitumor immunity in mice bearing TAL6 tumors. Our study demonstrates that a lipidated polyepitope cancer vaccine could be employed to generate potent anti-tumor immune responses, including humoral and cellular immunity, which could be beneficial in the treatment of TAL6^+^ cancer.

## 1. Introduction

Cancer vaccines represent a promising strategy to generate a protective immune response in order to recognize and eliminate malignant cells. In addition to the effective antitumor effects of cytotoxic T cells, antibodies have been proven to contribute their values to antitumor therapy as first-line drugs, such as trastuzumab (against HER2) and Avastin (against VEGFA), in the treatment of several cancer types [1]. The mechanisms of action of antibodies in inhibiting tumor growth include complement-dependent cytotoxicity and antibody-dependent cell-cytotoxicity (ADCC), where the tumor antigens are recognized by specific antibodies, and further can trigger the activation of natural killer cells and macrophages to destroy tumors. Thus, to achieve long-lasting and highly effective antitumor immunity via cancer vaccines, the enhancement of both humoral immunity induced by B cell epitopes and cellular immunity induced by cytotoxic T lymphocyte (CTL) epitopes is required.

B cell epitope-based peptide vaccines have been widely used in infectious diseases that can induce neutralizing antibody responses [2,3]. Moreover, peptide-based vaccines consisting of immunogenic epitopes are widely used in cancer vaccine development [4]. For example, HER2-derived peptide vaccines have been tested in human clinical trials and show promising outcomes when combined with trastuzumab [5]. The chimeric B cell epitope peptides of multiple receptor tyrosine kinases (HER-1, HER-3, IGF-1R and VEGF) incorporate a T cell epitope that elicits a polyclonal anti-tumor antibody response [6]. Several approaches have been developed to improve the immunogenicity of peptide vaccines, including combining them with different adjuvants and optimizing the delivery vectors. For example, bacteria-derived lipid-conjugated peptides can be recognized by the innate immune system, mainly through pattern recognition receptors, and induce a strong immune response. Thus, bacterial or synthetic lipids conjugated to antigens have shown adjuvant properties and are an ideal strategy for peptide-based cancer vaccine design [7]. Moreover, lipidated recombinant proteins have many advantages over traditional adjuvants, in terms of low toxicity and high efficacy in eliciting both mucosal and systemic immune responses [8,9].

Tumor-associated antigen L6 (TAL6), also known as transmembrane 4 L-six family member 1 (TM4SF1), is a small plasma membrane glycoprotein with four transmembrane domains and two extracellular loops (short EL1 and long EL2) [10]. TAL6 is highly expressed in several types of human cancer tissues, but minimally expressed in normal tissues [11,12,13], and its oncogenic roles in promoting cancer migration and angiogenesis [14,15] are associated with poor prognosis [13]. Therefore, TAL6 is an attractive target for cancer treatment. Based on the limited effects of TAL6-targeted antibodies in clinical trials [16], modified TAL6-targeted antibody-based strategies have been developed to improve the therapeutic efficacy, such as antibody-directed enzyme prodrug therapy [17], radioimmunotherapy [18], and conjugation with anti-tumor drugs [19]. In addition to these, synthetic peptide-based cancer vaccines against tumor antigens are easily tested clinically, and it is feasible to design polypeptide vaccines against one or several tumor-associated antigens (TAAs). For example, CTL epitopes of TAL6 [20], or a chimeric peptide containing both B and T cell epitopes of TAL6, have been developed and show promising therapeutic effects [21].

Transmembrane proteins could be difficult to express and purify due to their hydrophobic sequences [22]. Alternatively, expressing the extracellular domain of TAAs for immunization to induce antibody production is feasible. A vaccine design containing the large fragment of the extracellular domain of human tumor antigen HER2 has been proved to elicit both antibodies and cellular responses against HER2, and inhibit human breast tumor growth [23]. To extend this concept for generating effective protein vaccines, we construct a polypeptide composed with a long extracellular loop EL2 of TAL6, an identified B cell epitope of TAL6, and a universal Th cell epitope. Moreover, bacteria-derived lipids that activate Toll-like receptor 2 (TLR2) signaling are conjugated on the TAL6 polypeptides as intrinsic adjuvants to boost the response to vaccination. Here, we demonstrate that the addition of lipidation to a series of TAL6 epitopes will create a cancer vaccine that can trigger effective and protective antitumor immunity.

## 2. Materials and Methods 

### 2.1. Production of Recombinant Proteins rTh-Epi-L6 and Rlipo-Th-Epi-L6

**Figure 1:**
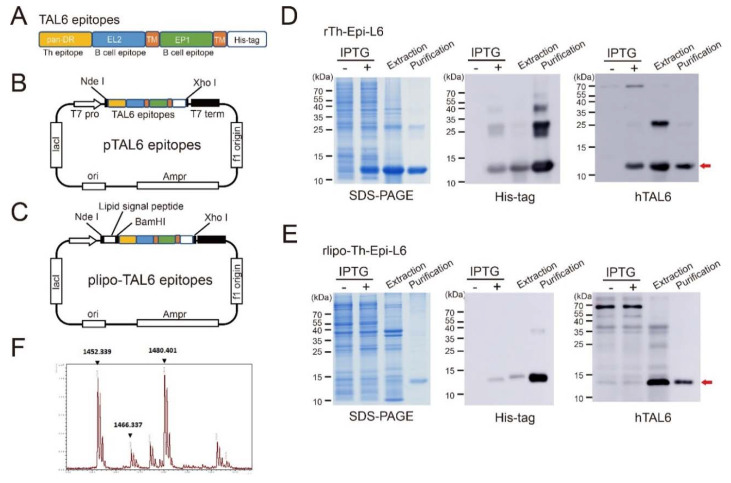
Characterization of recombinant non-lipidated (rTh-Epi-L6) and lipidated (rlipo-Th-Epi-L6) polyepitope vectors and proteins. (**A**) Epitopes of TAL6. The epitopes of TAL6 which trigger different immune cell response were sequentially combined with a HisTag sequence at the C-terminus. Pan-DR, helper T epitope; EL2, extracellular loop of TAL6; TM, transmembrane sequence (AAALLMLLPAFV); EP1, B cell epitope. (**B**) Plasmid pTAL6 was constructed by inserting the TAL6 epitopes into vector pET22b for production of the rTh-Epi-L6 protein. (**C**) Plasmid plipo-TAL6 was constructed by adding the D1 sequence to the pTAL6 vector for production of the rlipo-Th-Epi-L6 protein. (**D**) rTh-Epi-L6 expression and purification were monitored by reducing SDS-PAGE (left) and Western blotting with anti-HisTag (middle) and TAL6 (right) antibodies. (**E**) rLipo-Th-Epi-L6 expression and purification were monitored by reducing SDS-PAGE (left) and Western blotting using anti-HisTag (middle) and TAL6 antibodies (right). (**F**) Mass spectrometry analysis of rlipo-Th-Epi-L6. After the digestion of rlipo-Th-Epi-L6 with trypsin, the sample was analyzed by MALDI-TOF. The spectrum showed 3 peaks with m/z values of 1452, 1466 and 1480, corresponding to the expected mass differences for a lipidated peptide.

These polyepitopes of TAL6 were composed with helper T cell epitope (pan-DR), B cell epitope (EP1, aa114-133) [21], and parts of TAL6 regions outside the membrane containing extracellular loop 2 (EL2, aa 114-164), and were separated by short transmembrane (TM) sequence (AAALLMLLPAFV) for eliciting the antibodies (Figure 1A). The polyepitopes of TAL6 were constructed into the IPTG inducted expression vector to generate the plasmid of pTAL6 epitopes (Figure 1B). Previously, we identified a fusion sequence (D1, MKKLLIAAMMAAALAACSQEAKQEVKEAVQAVESDVKDTA) that can be fused with the nonlipidated immunogen to achieve high expression levels of the recombinant lipo-immunogen [24]. To generate lipidated polyepitopes of TAL6 (rlipo-Th-Epi-L6), a fusion sequence (D1) was inserted at the N-terminus of the TAL6 epitopes, and constructed into the IPTG-inducted expression vector to get the plasmid of lipo-TAL6 epitopes (Figure 1C). The synthetic nucleotide sequence of the TAL6 polyepitope gene with a hexahistidine tag (HisTag) sequence at the C-terminal end was cloned into the NdeI-Xho I sites of the expression vector pET-22b (+) (Novagen, Madison, WI), to generate a plasmid encoding the TAL6 epitopes (pTAL6 epitopes). The D1 domain and lipid signal peptide of the lipoprotein Ag473 were cloned into the NdeI and BamHI sites of pET-22b (+) to generate a plasmid encoding the lipidated TAL6 epitopes (plipo-TAL6 epitopes). The recombinant TAL6 polyepitope protein (rTh-Epi-L6) and recombinant lipidated TAL6 polyepitope protein (rlipo-Th-Epi-L6) were expressed and purified as previously described [13]. Briefly, the plasmid encoding rTh-Epi-L6 was transformed into *E. coli* strain BL21 (DE3) (Invitrogen, CA, USA), and 1mM IPTG was added when OD = 0.6 to induce protein expression, followed by an incubation at 20 °C for 18 h. The bacterial pellets were suspended in 50 mM Tris-Cl and 150 mM NaCl, pH 8.9 buffer. The rlipo-Th-Epi-L6 protein was induced with 1 mM IPTG at OD = 0.3 and incubated at 12 °C for 20 h after the transformation of the corresponding plasmid into the C43 (DE3) strain of *E. coli* [24]. The bacterial pellets were suspended in 50 mM Tris-Cl and 150 mM NaCl, pH 8.9 buffer. The purification of recombinant proteins was carried out as described in a previous study [25]. Briefly, proteins were extracted from the cell pellet in 6 M guanidine hydrochloride (GdnHCl) buffer, purified using immobilized metal affinity chromatography columns (QIAgen, Hilden, Germany), and further refined using an anion exchange column (Ni-NTA super flow; slurry). The purified proteins were then dialyzed against 10 mM dibasic sodium phosphate buffer. Because a level of endotoxin < 20 EU/mL for recombinant subunits was recommended [26], we set endotoxin < 10 EU/mg as our standard. The detection limit of endotoxin in our experiment was 10 EU/mg. The residual endotoxin concentration was below 10 EU/mg. Both proteins were analyzed by SDS-PAGE with Coomassie blue staining. 

### 2.2. Characterization of the Recombinant Proteins

Purified rTh-Epi-L6 and rlipo-Th-Epi-L6 were detected by an anti-His antibody (Bio-Rad, CA, USA) and a rabbit anti-human TAL6 antibody (Sigma, St Louis, MO, USA) by Western blotting. The lipidated N-terminal fragment of rlipo-Th-Epi-L6 was identified as previously described by matrix-assisted laser desorption ionization time-of-flight (MALDI-TOF) mass spectrometry (Bruker Daltonics GmbH, Leipzig, Germany) [13].

### 2.3. Activation of Bone Marrow-Derived Dendritic Cells (BM-DCs)

BM-DCs from C57BL/6 mice were assessed in culture as previously described [24]. Briefly, after 6 days in differentiation, BM-DCs (1 × 10^6^ cells/mL) were stimulated with lipopolysaccharide (LPS) (0.1 μg/mL), polymyxin B (PMB) (30 μg/mL) and 50 nM rTh-Epi-L6 or rlipo-Th-Epi-L6 for 24 h. The cell surface markers (CD40 and CD80) of BM-DCs were detected using flow cytometry (FACSCalibur, BD Biosciences, San Jose, CA, USA). The dendritic cells were gated as CD11c^+^/MHC II^+^ cells (Figure S1A). The cytokines secreted (TNF-α, IL-6 and IL12p40) from BM-DCs were measured using ELISA kits (Invitrogen, Carlsbad, CA, USA).

### 2.4. Antibody Isotypes Analysis

C57BL/6 mice were subcutaneously immunized three times at a 2-week interval with PBS, 50 μg rTh-Epi-L6 or 50 μg rlipo-Th-Epi-L6. Serum samples were collected at the indicated week after the first immunization. EP1-specific IgG, IgG1 and IgG2a titers in sera were measured by ELISA. Briefly, 10 μg/mL EP1 peptide was coated onto 96-well plates overnight at 4 °C. The mouse sera were diluted using a three-fold serial dilution (starting at 1:30) and incubated for 2 h at room temperature. Bound IgG was detected with HRP-conjugated goat anti-mouse IgG (Thermo Fisher Scientific, Waltham, MA, USA), HRP-conjugated goat anti mouse IgG1 (Zymed Laboratories Inc, South San Francisco, CA, USA) or HRP-conjugated goat anti mouse IgG2b and IgG2c (Zymed Laboratories Inc, South San Francisco, CA, USA), and was developed by using SureBlue TMB 1-Component Peroxidase Substrate (KPL). The absorbance was measured using an ELISA reader at 450 nm.

### 2.5. T Cell Response Assay

The TAL6 polyepitopes’ immunization in C57BL/6 mice was performed as previously described [20]. Briefly, 50 μg rTh-Epi-L6 and 50 μg rlipo-Th-Epi-L6 were immunized by footpad injection three times at one-week intervals. The splenocytes and lymphocytes from lymph nodes were collected 7 days after the last boost and restimulated with 10 μg/mL of pan-DR or SSC peptide for 48 h. The T cell responses were assessed by an IFN-γ ELISPOT assay as previously described [25]. The spots were stained using 3-amine-9-ethyl carbazole (Sigma-Aldrich, St. Louis, MO, USA) and counted by an ELISPOT reader (Cellular Technology Ltd., Shaker Heights, OH, USA). For the cytokines secretion assay, splenocytes were plated at a density of 5 × 10^5^ cells per well in 24-well plates. The cells were stimulated with pan-DR (10 μg/mL) for 4 days. The culture supernatant was harvested and the levels of IFN-γ, IL-5 and IL-17A were analyzed using ELISA Kit (Invitrogen, Carlsbad, CA, USA). The absorbance was measured using an ELISA reader at 450 nm.

### 2.6. In Vivo Tumor Protection Experiments

Six- to eight-week-old female C57BL/6 mice were subcutaneously immunized 3 times with 50 μg rTh-Epi-L6 or 50 μg rlipo-Th-Epi-L6 at 2-week intervals. PBS was used as well. At 14 days after the last injection, the EL4/L6 cells (1 × 10^5^) that stably express TAL6 were inoculated subcutaneously on the opposite site of the protein injection. Tumor sizes were measured 3 times per week in orthogonal dimensions by using an electronic caliper. Tumor volume was calculated with the following formula: (length × width^2^)/2. The protocol was approved by the Institutional Animal Care and Use Committee of the National Health Research Institutes (NHRI).

### 2.7. Statistical Analysis

All statistical tests were performed to compare the differences between mean values of the groups using two-sided Student’s *t*-tests. Statistical significance was considered at *p* < 0.05.

## 3. Results

### 3.1. Characterization of Purified Recombinant Proteins Composed with Polyepitopes of TAL6

To activate the humoral and cellular immunity against tumor antigens by vaccination, we designed the polyepitopes, which are recombinant proteins constituted by multiple epitopes of TAL6, and a HisTag was included at the C-terminus of the multiple epitopes for purification (Figure 1A–C). The recombinant polyepitopes of TAL6 (rTh-Epi-L6) were produced from the plasmid of pTAL6 epitopes with IPTG induction in the *E. coli* system, and then purified using an immobilized metal affinity chromatography (IMAC) column (Figure 1D). The rTh-Epi-L6 proteins were identified by anti-HisTag and anti-TAL6 antibodies in a Western blot (Figure 1D). The lipidated polyepitopes of TAL6 (rlipo-Th-Epi-L6) were produced from the plasmid of plipo TAL6 epitopes, and characterized with a strategy similar to non-lipidated proteins (Figure 1E). The lipid moiety at the N-terminus of the rlipo-Th-Epi-L6 proteins was determined by peptide mass fingerprinting (PMF), and identified three major peaks with masses of 1452, 1466 and 1480.4 Da in the mass spectral data derived from rlipo-Th-Epi-L6 proteins (Figure 1F). Given that the distance between the major peaks was approximately 14 Da, which is the predicted mass of the CH2 structure [25], the spectrum of these major peaks was considered the signature of the lipid modifications in rlipo-Th-Epi-L6 proteins. The results demonstrate that the purified polyepitopes and lipidated polyepitopes of TAL6 have corrected protein size and lipid moiety.

### 3.2. Rlipo-Th-Epi-L6 Proteins Activate BM-DCs through TLR2

**Figure 2:**
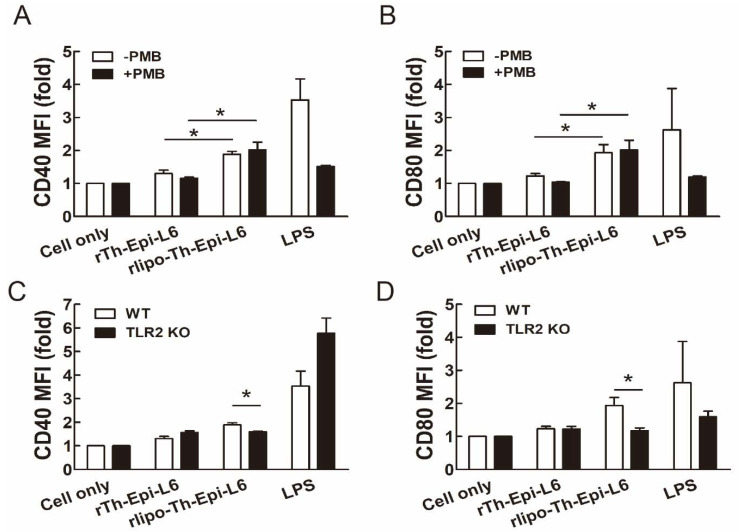
rLipo-Th-Epi-L6 activates bone marrow-derived dendritic cells (BM-DCs). (**A**,**B**) BM-DCs from wild-type mice were cultured in medium supplemented with rTh-Epi-L6 (50 nM), rlipo-Th-Epi-L6 (50 nM), or LPS (0.1 μg/mL), in the presence or absence of polymyxin B (PMB; 30 μg/mL). After a 24-h incubation, the expression of the DC surface markers CD40 (**A**) and CD80 (**B**) was analyzed using flow cytometry. The experiments were performed in triplicate, and the mean fluorescence intensity (MFI) of the cells cultured in medium alone was defined as 1. (**C**,**D**) BM-DCs isolated from wild-type and TLR2 knockout (TLR2 KO) mice were cultured in medium supplemented with rTh-Epi-L6 (50 nM), rlipo-Th-Epi-L6 (50 nM) or LPS (0.1 μg/mL), and then DC surface markers CD40 (**C**) and CD80 (**D**) were analyzed using flow cytometry. Data are presented as the means ± SEM of at least three independent experiments. * *p* < 0.05 by *t*-test.

First, we determined whether the purified polyepitope proteins have immunostimulatory effects on mouse BM-DCs. The endotoxin LPS levels of the purified proteins were lower than 10 EU/mg, and polymyxin B (PMB), which was used to clear endotoxin contamination, was added to eliminate residual LPS. We found that rlipo-Th-Epi-L6 was capable of stimulating the expression of the costimulatory molecules CD40 and CD80 on BM-DCs, while rTh-Epi-L6 was ineffective (Figure 2A,B and Figure S1B). PMB addition had no effect on the levels of CD40 and CD80 expression on BM-DCs stimulated with rlipo-Th-Epi-L6, indicating that the DC activation by rlipo-Th-Epi-L6 was not caused by residual LPS. To further investigate whether rlipo-Th-Epi-L6 stimulated BM-DC activation through TLR2, we compared the costimulatory molecules CD40 and CD80 on BM-DCs isolated from wild-type mice and TLR2 knockout (KO) mice. The elevated levels of the costimulatory molecules CD40 and CD80 on wild-type BM-DCs, stimulated with rlipo-Th-Epi-L6, were significantly decreased on TLR2 KO BM-DCs (Figure 2C,D and Figure S1C).

**Figure 3:**
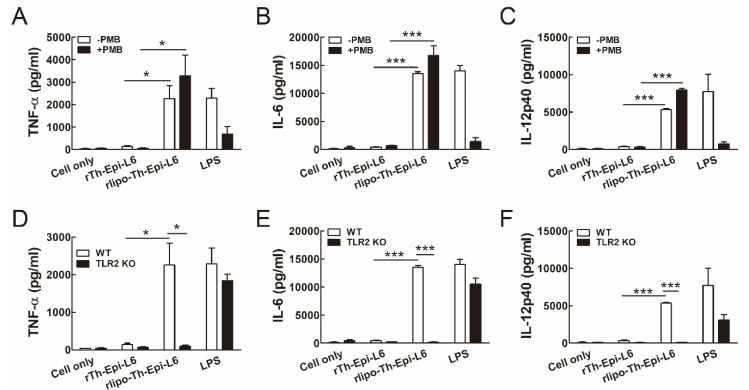
rLipo-Th-Epi-L6 stimulates bone marrow-derived dendritic cells (BM-DCs) to secrete cytokines. (**A**–**C**) Cytokine TNF-α (**A**), IL-6 (**B**), and IL-12p40 (**C**) secretion by DCs was stimulated by rlipo-Th-Epi-L6. BM-DCs were cultured with rTh-Epi-L6 (50 nM), rlipo-Th-Epi-L6 (50 nM), or LPS (0.1 μg/mL) in the presence or absence of polymyxin B (PMB; 30 μg/mL). After a 24-h incubation, the supernatants were harvested and analyzed for cytokine production by ELISA. (**D**–**F**) Cytokine TNF-α (**D**), IL-6 (**E**) and IL-12p40 (**F**) secretion by DCs isolated from wild-type and TLR2 knockout (TLR2 KO) mice was stimulated by rlipo-Th-Epi-L6. Data are presented as the means ± SEM of at least three independent experiments. * *p* < 0.05, *** *p* < 0.001 by *t*-test.

Upon maturation, DCs can secrete several cytokines for T cell immunity. We found that the secretion of TNF-α, IL-6 and IL-12p40 from BM-DCs was induced by rlipo-Th-Epi-L6 with or without PMB addition, but not by rTh-Epi-L6 (Figure 3A–C). These results indicate that the activation of BM-DCs by rlipo-Th-Epi-L6 was not due to any residual LPS, and that the lipidated portion of the protein was responsible for the effect. Moreover, the secretion of these cytokines from BM-DCs was stimulated by rlipo-Th-Epi-L6 in wild-type mice, but not in TLR2 KO mice (Figure 3D–F). Since previous results have demonstrated that the amino acid sequence of the D1 domain (the fusion partner for recombinant lipoprotein production) is unable to stimulate the production of cytokines [24], these data clearly demonstrate that the lipidated form of the TAL6 polyepitope protein is able to activate DCs through TLR2, by stimulating the expression of costimulatory molecules and cytokines.

### 3.3. Immunization with Rlipo-Th-Epi-L6 Increases Antigen-Specific Antibody Levels and T Helper Cell Responses

**Figure 4:**
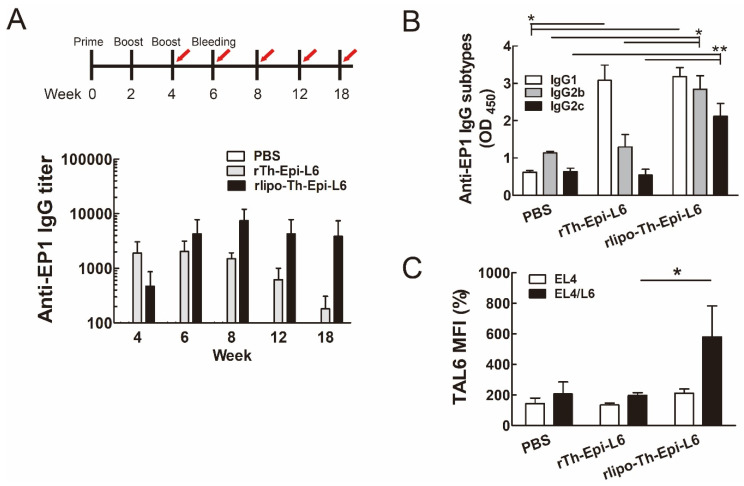
Immunization with rlipo-Th-Epi-L6 induced higher levels of anti-Ep1 antibody and Th1 immunity. (**A**) The immunization schedule in C57BL/6 mice (*n* = 6, per group) involving 3 injections (1 prime and 2 boosts) at 2-week intervals. Serum samples were collected from immunized mice at the indicated time points (red arrows) after the first immunization. Anti-EP1 IgG titers were determined using ELISA. (**B**) The subtypes of anti-EP1 IgG were determined. (**C**) The ability of immunized mouse serum to recognize TAL6 on cancer cell surface, depicted as the mean fluorescence intensity (MFI). The data are presented as the mean ± SEM of triplicate samples. * *p* < 0.05, ** *p* < 0.01 by *t*-test.

Next, we examined whether antigen-specific antibodies were induced in mice immunized with TAL6 polyepitope proteins. To evaluate the level of TAL6 antigen-specific antibodies, we detected the antibody titer recognizing the EP1 epitope. Both TAL6 polyepitope proteins stimulated the production of EP1 antibodies, but rlipo-Th-Epi-L6 induced higher levels of EP1 antibodies for longer periods than rTh-Epi-L6 (Figure 4A). Moreover, rlipo-Th-Epi-L6 immunization induced higher IgG2b and IgG2c levels than rTh-Epi-L6 immunization, but induced similar IgG1 levels (Figure 4B), suggesting high levels of ADCCs could be generated [27]. To investigate whether the antibodies produced by mice immunized with TAL6 polyepitope proteins can specifically recognize the TAL6 antigen, the mouse serum was used to stain the TAL6 antigen on the surface of tumor cells. The serum from the rlipo-Th-Epi-L6 group showed a stronger signal in recognizing the surface TAL6 proteins of EL4/L6 cells, while there was no elevated signal in the control EL4 cells, indicating the specificity of the induced antibodies (Figure 4C). 

**Figure 5:**
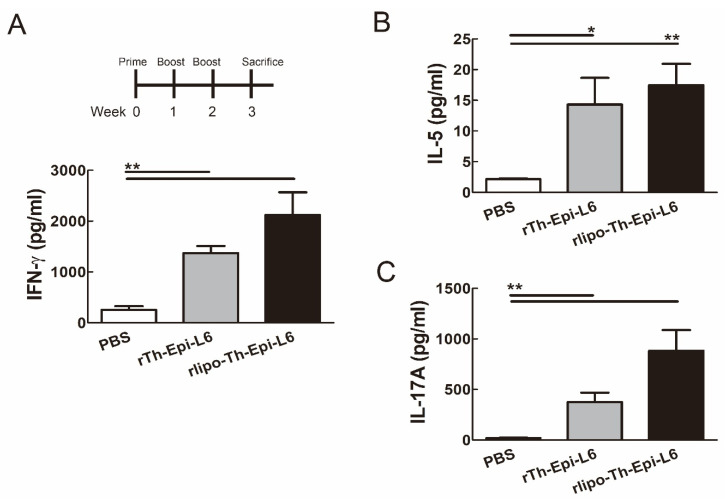
Immunization with rlipo-Th-Epi-L6 protein induced higher levels of cytokine release. (**A**–**C**) The immunization schedule in C57BL/6 mice (*n* = 6, per group) involving injection of recombinant proteins 3 times at 1-week intervals. Seven days after the final immunization, mice were sacrificed, and splenocytes were isolated from the spleens and plated at a density of 5 × 10^5^ cells/well in 24-well plates. The cells were incubated with 10 μg/mL pan-DR peptides for 4 days and then analyzed for secreted cytokines. The supernatants were collected, and the levels of IFN-γ (**A**), IL-5 (**B**), and IL-17A (**C**) were measured by ELISA. Bars and error bars show the mean ± SEM. * *p* < 0.05, ** *p* < 0.01 by *t*-test.

To assess whether T helper immunity was elicited in mice immunized with TAL6 polyepitope proteins, we measured the secreted cytokines from T helper cells isolated from the spleens of immunized mice. With pan-DR peptide stimulation, the secretion of IFN-γ, IL-5 and IL-17A was increased by immunization with either of the two polyepitope proteins, but rlipo-Th-Epi-L6 induced moderately higher levels of cytokine secretion than rTh-Epi-L6 (Figure 5). Thus, the results demonstrated that the lipidated polyepitope protein has better immunogenicity than the nonlipid polyepitope protein.

### 3.4. Immunization with Rlipo-Th-Epi-L6 Induces Antigen-Specific T Cell Responses

**Figure 6:**
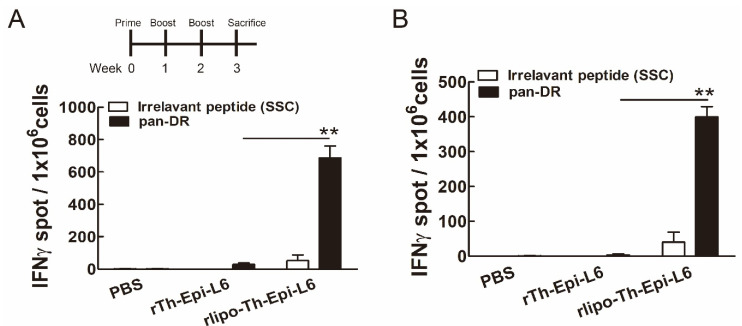
Immunization with rlipo-Th-Epi-L6 induced higher levels of pan-DR-specific IFN-γ-secreting cells. (**A**,**B**) The immunization schedule in C57BL/6 mice by injecting indicated recombinant proteins 3 times at 1-week intervals. *n* = 6, each group. (**A**) Splenocytes (5 × 10^5^ cells/well) or (**B**) lymph node cells (2 × 10^5^ cells/well) from immunized mice were incubated with irrelevant control peptide (SSC) or pan-DR peptides for 48 h in an anti-IFN-γ-coated 96-well ELISPOT plate. The IFN-γ spots were measured using an ELISPOT reader. Bars and error bars show the mean ± SEM. ** *p* < 0.01 by *t*-test.

We then investigated whether rlipo-Th-Epi-L6 immunization can induce antigen-specific T cell responses. Seven days after the mice were immunized with the TAL6 polyepitope proteins, their splenocytes were harvested and stimulated with pan-DR peptide to assess the T cell responses using an IFN-γ ELISPOT assay. Immunization with rlipo-Th-Epi-L6 produced a higher number of IFN-γ-secreting cells than immunization with rTh-Epi-L6 after pan-DR stimulation (Figure 6A). However, the number of IFN-γ-secreting cells was not significantly increased with irrelevant control peptide (SSC, SSCSSCPLSK) stimulation. Consistently, an elevated number of IFN-γ-secreting cells was detected in the lymph node (inguinal region) cells with pan-DR epitopes stimulation (Figure 6B). These results demonstrated that T cell responses can be induced using rlipo-Th-Epi-L6 immunization.

### 3.5. Immunization with Rlipo-Th-Epi-L6 Reduces Tumor Size and Provide the Protective Effect in the Disease-Free Survival

**Figure 7:**
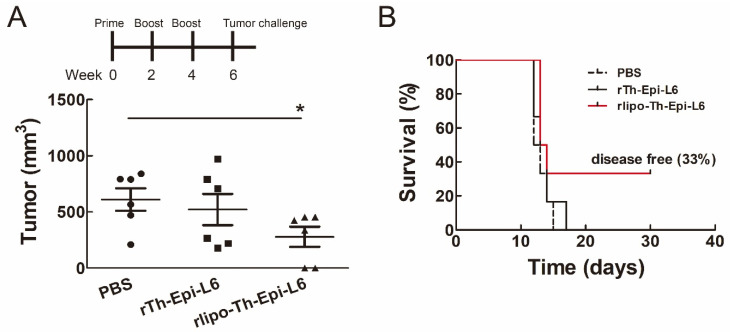
Immunization with rlipo-Th-Epi-L6 inhibited tumor growth and prolonged disease-free survival time. (**A**) C57BL/6 mice were immunized (*n* = 6, per group) by injecting the indicated recombinant proteins 3 times at 2-week intervals, and then they were subcutaneously inoculated with EL4/L6 thymoma cells (1 × 10^5^/mouse). The tumor volumes were measured 3 times per week. The graphs show the mean ± SEM on day 12 after tumor challenge. * *p* < 0.05 by *t*-test. (**B**) The disease-free survival rates were analyzed.

To evaluate the protective effect of the TAL6 polyepitope proteins in vivo, mice were immunized via three injections and then subcutaneously injected with EL4/L6 thymoma cells. The tumors were significantly smaller in the mice immunized with rlipo-Th-Epi-L6, compared to those in the PBS or rTh-Epi-L6 groups on day 12 after tumor challenge (Figure 7A). Moreover, at the experimental end point of 30 days, more mice immunized with rlipo-Th-Epi-L6 (33%) remained disease-free than in the other groups (0%) (Figure 7B). These results demonstrated that immunization with rlipo-Th-Epi-L6 provides increased prophylactic efficacy compared to non-lipidated rTh-Epi-L6.

## 4. Discussion

**Figure 8:**
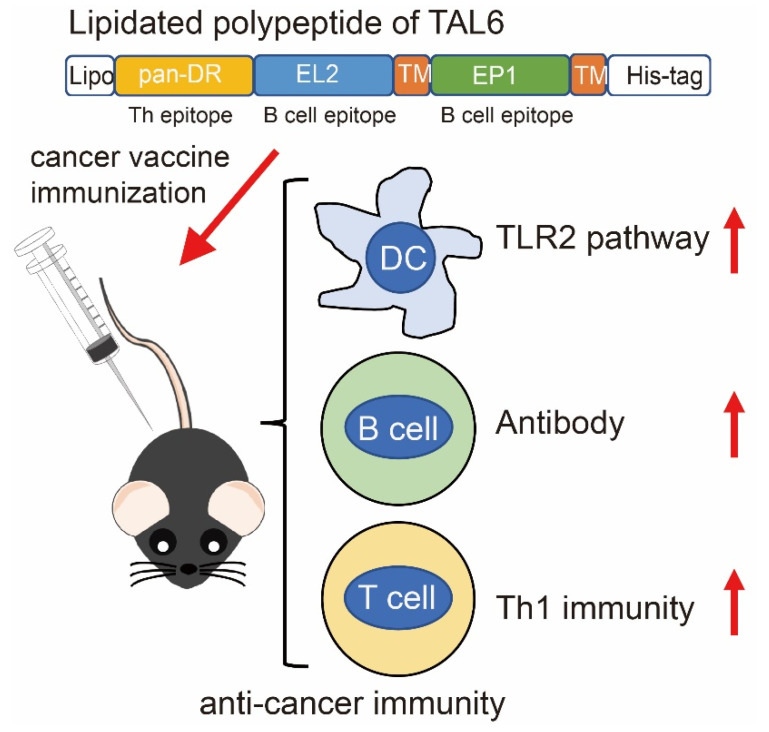
Mode of action of the lipidated polypeptide of TAL6. This cancer vaccine immunization elicits anti-cancer immunity, including TLR 2 signaling for DC activation, antigen-specific antibody production, and Th1 immunity.

In this study, a lipidated TAL6 polyepitope protein that elicits both cellular (Th1) and humoral (Th2) immune responses was developed. We demonstrated that rlipo-Th-Epi-L6 optimally stimulated TLR2 signaling and further activated BM-DCs. Mice vaccinated with the lipidated polyepitope protein exhibited T helper 1 cell immunity for antigen-specific antibody production. Moreover, this vaccination induced antigen-specific T cell responses. Combining the effects of both humoral and cellular immune responses, this vaccination strategy reduced tumor size in mice and prolonged disease-free survival (Figure 8).

Antibody induction to recognize TAAs plays critical roles in triggering the immune system to destroy the cancer cells with the antigen, and has proven to be a useful strategy in the treatment of cancer. However, multiple administrations of TAA vaccines are usually required to produce the therapeutic effect, and mutations in the TAA may lead to therapeutic unresponsiveness. Vaccination can induce long-term anti-tumor immunity by periodic boosting. It has been reported that polyclonal antibodies induced by vaccination to recognize different epitopes of HER2, a membrane-bound receptor for tumor growth, can have multiple effects on HER2 function, and efficiently decrease the seeding rate of HER2-dependent tumor xenografts [28]. Pathogenic membrane-bound receptors are suitable for use in vaccines that induce a polyclonal humoral immune response for mediating both ADCC and complement-dependent cytotoxicity in cancer treatment.

Studies of vaccines against the severe acute respiratory syndrome coronavirus (SARS-CoV) have redoubled since the beginning of the SARS-CoV-2 pandemic in 2019, and some of the lessons learned are applicable to cancer vaccines. For instance, we know that the receptor-binding domain (RBD) of the virus’ spike protein is the major immunogen for SARS-CoV vaccines [29]. An extended loop on the RBD is responsible for receptor binding [30], and this highly variable region is likely the determinant of differential receptor usage by different coronaviruses [31]. This suggests that long loops in transmembrane receptors may possess heightened antigenicity. TAL6 is a membrane-bound protein with four transmembrane domains and two extracellular domains, one of which contains an extended loop [10]. In this study, we have included the long extracellular loop 2 of TAL6 in the recombinant polyepitope proteins, with the goal of triggering additional humoral immunity in mice upon vaccination. The major limitation of this design is that the conformational structure could not be easily expressed in *E. coli*’s expression system. The other minor limitation is the *E. coli* expression system, which lacks glycosylation. Thus, B cell epitopes containing glycosylated sites could not be used in this design. In general, our approach would be more suitable for linear B cell epitopes without glycosylation.

Optimal adjuvants, called immunomodulators, are used to invoke a satisfactory immune response during vaccination [32]. They are small molecules that enhance the immunogenicity of vaccines, such as potassium alum. Most studies use incomplete Freund’s adjuvant or Montanide ISA-51 and other compositions to stimulate immune responses. Bacterial components, such as cholera toxin and *E. coli* heat-labile enterotoxin, can also be used as adjuvants to boost the mucosal vaccination against *H. pylori* in mice [33], but enterotoxicity limits their clinical applications. Outer membrane vesicles of *H. pylori* were reported as non-toxic adjuvants to induce long-term protection with humoral and Th-1 immunity against *H. pylori* in mice [34]. Lipids are one component of the outer membrane vesicles of Gram-negative bacteria, and play roles in immune regulation [35]. Different lipid formulations have been developed as adjuvants to stimulate antigen-specific IgG production, such as the glucopyranosyl lipid adjuvant for the Ebola virus-like particle vaccine in mice [36]. Liposomes containing three adjuvants (polyinosinic-polycytidylic acid, host defense peptide IDR-1002, and polyphosphazene) enhanced mucosal immunity in mice [37]. These studies show that lipid-based adjuvants indeed boost strong humoral immunity in mice.

The major potential benefit of our vaccine strategy is the induction of both T cell and polyclonal antibody responses. Our previous studies showed that recombinant lipidated proteins containing domain 3 of the envelope protein of dengue virus can enhance both antibody titers and neutralizing antibody titers [38,39]. Similar effects were observed with lipidated domain 3 of the envelope protein of the Zika virus [40]. We previously identified the B cell epitope of TAL6 that can induce ADCC to kill cancer cells [21]. In this report, we demonstrated that the lipidated protein containing B cell epitopes of TAL6 and pan-DR can increase anti-tumor effects. The lipidated polyepitope protein can induce higher levels of IFN-γ secretion, but the non-lipidated counterpart cannot. The polarization of the immune response towards the Th1-type immune response plays an important role in controlling tumor growth. This study presents a feasible vaccination strategy of combining epitopes for humoral and cellular immunity against TAL6 in a lipidated protein to prevent the growth of TAL6-positive tumors.

## Supplementary Materials

The following are available online at https://www.mdpi.com/2076-393X/8/4/620/s1, Figure S1: Supplemental Figure S1, related to Figure S2. (A)The cultured bone marrow cells were collected and analyzed using flowcytometry. SSC vs. FCS density plot was gated for anti-CD11c and anti-MHC class II analysis. The CD11c+/MHC II+ cells were indicated as dendritic cells. (B) Representative flow cytometry analysis for Figure S2A,B. (C) Representative flow cytometry analysis for Figure S2C,D.
