# Supplementary material for: A Polypeptide of Tumor-Associated Antigen L6 with Intrinsic Adjuvant Activity Enhances Antitumor Immunity
Source: Vaccines (Basel). 2020 Oct 21;8(4):620. doi: 10.3390/vaccines8040620 (PMC7711899; doi:10.3390/vaccines8040620)
Supplement: Supplementary file 1 [file vaccines-08-00620-s001.pdf]

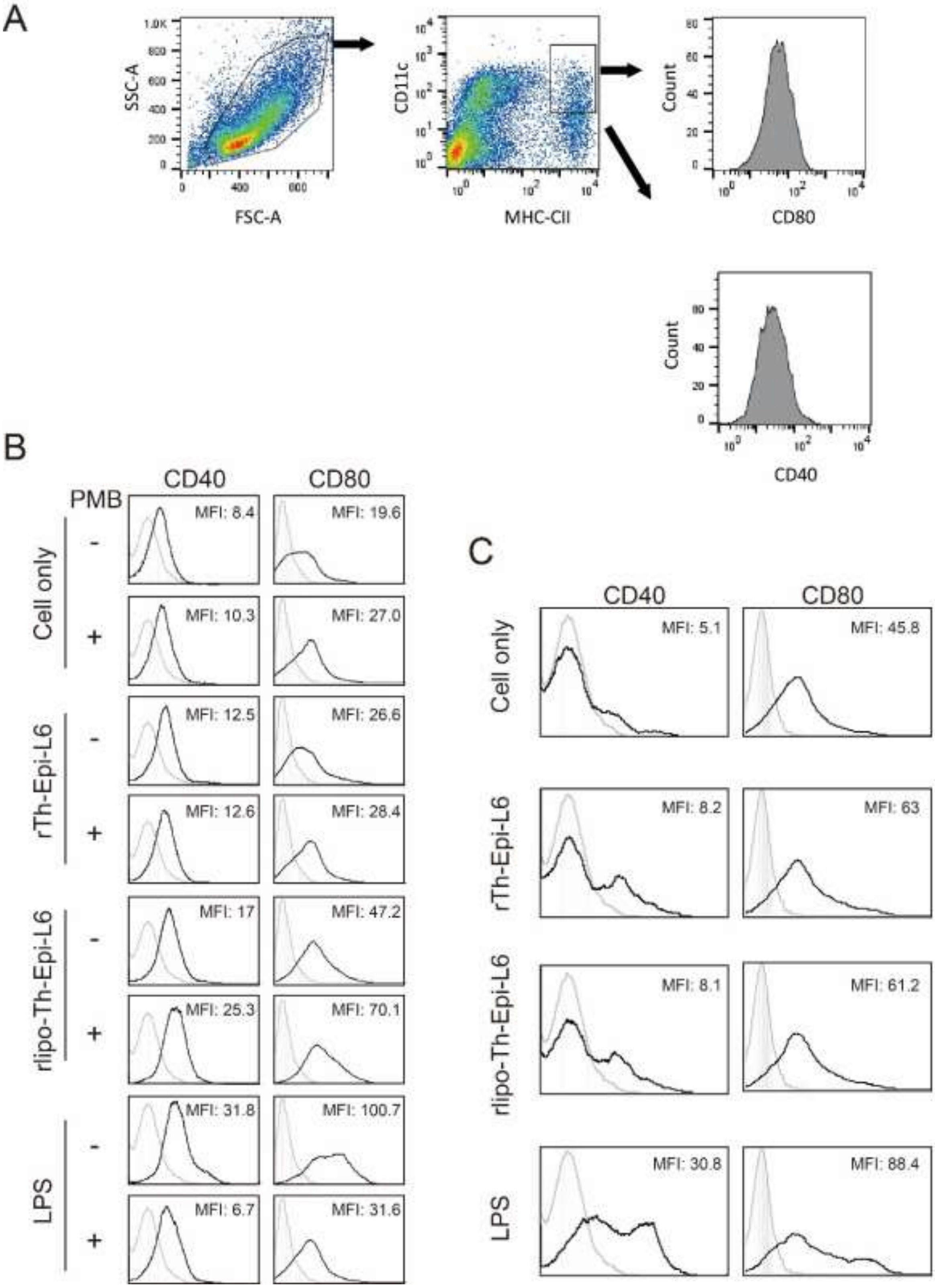

Figure S1. : Supplemental Figure 1, related to Figure 2. (A)The cultured bone marrow cells were. collected and analyzed using flowcytometry. SSC vs FCS density plot was gated for anti- CD11c and anti - MHC class II analysis. The CD11c+/MHC II+ cells were indicated as dendritic cells. (B)

Representative flow cytometry analysis for Figure 2A-B. (C) Representative flow cytometry analysis for Figure 2C-D.
